# Research Progress of a Potential Bioreactor: Duckweed

**DOI:** 10.3390/biom11010093

**Published:** 2021-01-13

**Authors:** Gui-Li Yang, Dan Feng, Yu-Ting Liu, Shi-Ming Lv, Meng-Meng Zheng, Ai-Juan Tan

**Affiliations:** 1 College of Life Sciences, Guizhou University, Guiyang 550025, China; glyang3@gzu.edu.cn (G.-L.Y.); fengdan1901@163.com (D.F.); liuyut90@163.com (Y.-T.L.); zmm18073198235@163.com (M.-M.Z.); 2 Key Laboratory of Conservation and Germplasm Innovation of Mountain Plant Resources, Ministry of Education, Guiyang 550025, China; 3 College of Animal Science, Guizhou University, Guiyang 550025, China; lvlvsm@163.com

**Keywords:** duckweed, genetic transformation, bioreactor, biosynthesis, chassis plant

## Abstract

Recently, plant bioreactors have flourished into an exciting area of synthetic biology because of their product safety, inexpensive production cost, and easy scale-up. Duckweed is the smallest and fastest-growing aquatic plant, and has advantages including simple processing and the ability to grow high biomass in smaller areas. Therefore, duckweed could be used as a new potential bioreactor for biological products such as vaccines, antibodies, pharmaceutical proteins, and industrial enzymes. Duckweed has made a breakthrough in biosynthesis as a chassis plant and is being utilized for the production of plenty of biological products or bio-derivatives with multiple uses and high values. This review summarizes the latest progress on genetic background, genetic transformation system, and bioreactor development of duckweed, and provides insights for further exploration and application of duckweed.

## 1. Introduction

Recently, plants have been considered as alternative bioreactors and have burgeoned the field of synthetic biology due to advantages such as product safety, inexpensive production cost, and easy scale-up [1,2,3]. Plant bioreactors are referred to as “chemical factories”, in which genetically modified crops are cultivated to produce biological agents with potential commercial values, such as vaccine antigens, antibodies, nutritional supplements, and industrial enzymes [4]. In the past two decades, some effective expression systems in plants have been widely investigated, and more than 100 recombinant proteins have been produced using diverse plant tissues [5]. For example, cholera toxin B subunit was transferred into potatoes that worked as edible vaccines [6,7]. Marquetblouin et al. [8] obtained the immunodominant antigen of measles virus by transforming it into carrots. Based on these results, transgenic plants might serve as effective expression systems for the production of recombinant proteins in the biopharmaceutical industry [9].

In-depth knowledge of plant molecular biology and gene engineering has led to the production of protein products using “naturalized bioreactors”. A multitude of plants have been utilized to produce recombinant proteins, such as tobacco [10], tomato [11], rice [12], and potato [13]. However, most of them are crops or terrestrial plants that compete with crops for land. Besides, genetically modified plants tend to cause gene drift, which is a great challenge for the industrialization of plant expression systems [14]. Therefore, the selection of a synthetic platform is essential to avoid food crisis and for maintaining the effective yield of recombinant products.

Duckweed is a tiny aquatic plant, which is easy to survive and widely distributed. The exponential growth of duckweed plays a vital role in the rapid production of proteins, resulting in a shorter production cycle [15]. Under certain conditions, the asexual reproduction of duckweed makes it genetically stable without genetic drift. Other desirable characteristics of large-scale production are simple and easy culture conditions [16]. In addition, duckweed is a non-crop plant that probably does not cause a food crisis. Therefore, duckweed could serve as a potential recombinant protein expression system. Dr. Anne-Marie Stomp of North Carolina State University was the first to propose the use of genetically modified duckweed as a carrier to produce recombinant proteins, which is still extensively researched [16,17,18].

There were a few review articles focused on duckweed. Xu et al. [19] summarized the research status about cultivation of high-starch duckweed and its biofuels conversion. Wang et al. [20] and An et al. [21] conducted a molecular analysis of duckweed genomics and transcriptomics. Appenroth et al. [22] reviewed the identification methods of duckweed genotypes that reliably and rapidly differentiated duckweed species. Although Ekperusi et al. [23] summarized the role of duckweed in phytoremediation, the review was devoid of a fundamental discussion about applying the duckweed as a potential bioreactor. Stomp [16] summarized the potential of duckweed several years ago as a valuable plant for biomanufacturing, though, numerous studies have emerged since then. Therefore, we review the inherent characteristics, genetic background, and genetic modification of duckweed to provide an insight into the latest progress of bioreactor applications of duckweed.

## 2. Biological Characteristics of Duckweed

### 2.1. Inherent Characteristics of Duckweed

**Figure 1:**
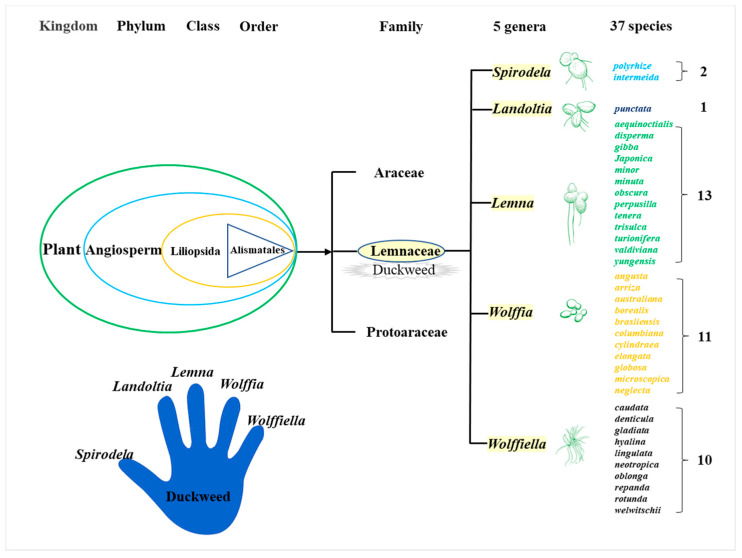
Lemnaceae family [22,26]. Drawn from representative plates in Reference [21].

**Figure 2:**
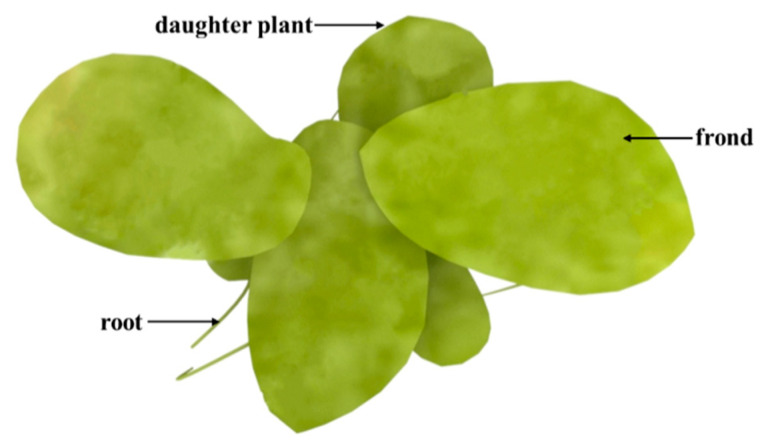
Schematic diagram of *Landotia punctata*.

Duckweed is a monocotyledon, belonging to the Lemnaceae family, which consists of five genera (*Spirodela*, *Landoltia*, *Lemna*, *Wolffia*, and *Wolffiella)* and 37 species (Figure 1) [24]. Duckweed is the smallest flowerer globally, with a size of only a few millimeters. The biological structure of duckweed is simple, which is always bilobed, obovate, or elliptic [22]. Duckweed usually reproduces asexually with an extremely short cycle. The daughter plants of the duckweed are produced from the budding pouch of the mother plant (Figure 2). The exponential reproduction of duckweed results in a high biomass growth rate. Duckweed is able to adapt to a wide range of pH, and the optimum pH for growth is 4.5~7.2. Duckweed can also survive at temperatures ranging from 2 to 35 °C, with an optimum temperature of 25 °C for growth. These properties contribute to its wide distribution in natural water bodies. It grows in paddy fields, ponds, lakes, and other static waters [24,25].

### 2.2. Culture Conditions of Duckweed

For the preservation of germplasm resources, the pure culture of whole duckweed plants has been established [27]. Duckweed can grow on a variety of carbon sources supplied with essential nutrients, although different growth rates are achieved [28]. Sucrose or other sugars supplemented with the medium at concentrations of 0.5%~3% and periodic or continuous artificial light provides duckweed with vigorous growth [29]. The length and replenishment time of the photoperiod differ from species to species of duckweed and depend on the intensity of the light provided. Duckweed prefers stationary culture conditions because the mechanical agitation of the medium deteriorates its growth. The optimum temperature for the laboratory culture also varies among species; however, growth at room temperature is economical for its cultivation [16]. Therefore, high biomass/unit time of genetically homogeneous duckweed can be produced by using appropriate media under the confined environment.

## 3. Genetic Background of Duckweed

The study of the duckweed genome is important for the comprehensive understanding of the composition and regulation of its genes. It is also essential for modifying duckweed at the molecular level so that it could be used as a bioreactor to synthesize biological products with high values.

### 3.1. Chloroplast Genome of Duckweed

Mardanov et al. [30] first reported the entire nucleotide sequence in the chloroplast genome (cpDNA) of duckweed (*Lemna minor*). Typically, cpDNA is 165,955 bp in length, which contains two inverted repeat regions (31,223 bp in length) separated by a small region (89,906 bp in length) and a large region (13,603 bp in length), both of which are single-copy regions. Wang et al. [31] produced cpDNA of *Woffiella lingulata*, *Woffia australiana*, and *Spirodela polyrhiza* to understand the genetic evolution among the duckweed subfamily members and used *Lemna minor* as the control. This study found that the chloroplast genomes of different genera were similar in gene composition and structure, implying that the gene content is conserved in duckweeds. Another finding of this study showed that rapid nucleotide substitutions and abundant insertions and deletions explained the cpDNA evolution of duckweed. In 2017, Ding et al. [32] completed the assembly of the cpDNA of *Landoltia punctata* by filtering genomic data and directly obtained the sequence from the extracted cpDNA. The comparison showed that *Landoltia punctata* cpDNA size obtained by both the methods was 171,013 bp, and the sequence similarity was 100%. In 2020, Zhang et al. [33] completed the assembly of cpDNA of *Spirodela polyrhiza* 7498 by the third generation of PacBio. The annular chloroplast genome with a length of 168,956 bp contains two 31,844 bp reverse repeats, one 91,210 bp single copy, together with one 14,058 bp single copy. A total of 107 unique genes were detected, among which, 78 were encoded proteins, 25 were tRNA genes, whereas 4 were rRNA genes. In comparison with the earlier version [31], the current version has improved the quality and integrity of short reads; in particular, two repeated fragments were retrieved in the ycf2 gene.

Chloroplast genomes of all genera of duckweed have been reported (Table 1). The sizes of cpDNA within some subfamilies of higher plants were reported as being conservative, such as *Maloideae,* which has a similar cpDNA size between *Pydus spinosa* (159,161 bp) and *Malus pdunifolia* (160,041 bp) [34]. Similarly, cpDNA sizes of different duckweed are less variable, with a length range of 165,955 to 171,103 bp. All of them include around 31 Kb length inverted repeats, accounting for the variation in the size of cpDNAs of duckweed, which is consistent with other plants [35,36]. Comparative analysis indicated that the cpDNA of other duckweed was conserved in gene number and organization with *Lemna minor* [30]. However, compared with the cpDNA of other grass families, substantial variations involved nucleotide insertions, deletions, and substitution in non-coding regions of duckweed [31]. The cpDNA of duckweed can serve as a complicated single-locus barcode, as other plants used in the integrative analysis. Developing the chloroplast transformation system for the application of duckweeds in the industry is of great importance.

### 3.2. Mitochondrial Genome of Duckweed

The plant cell contains three genomes under normal conditions, including nuclear (nDNA), chloroplast, and mitochondrial genomes (mtDNA) [37]. Of these, the mtDNA serves as the primary center for Adenosine triphosphate (ATP) production through oxidative phosphorylation, which has a vital role in plant metabolism and growth [38]. Besides, mitochondria in the plant can initiate a new strategy for the highly expressed transgenes because they are maternally inherited. In 2012, Wang et al. [39] reported the mitochondrial genome of duckweed *Spirodela polyrhiza*, which is a highly packed mitochondrial genome among monocotyledons and consists of 228,493 bp in length. In total, 57 genes encoded 19 tRNAs (tRNA consensus amino acids) that recognized 15 amino acids, 35 known proteins, and 3 rRNAs. Sequence analysis showed that 4.1% of the mtDNA stems from cpDNA, whereas a few nDNAs were found in the mtDNA. In addition, the phylogenetic tree indicated no synteny in mtDNA between *Spirodela* and rice, although *Spirodela* shared a common ancestor with other monocots. This is the only report about the mitochondrial genome of duckweed.

### 3.3. Whole-Genome Sequencing of Duckweed

In 2014, Wang et al. [40] first reported the whole-genome sequence of *Spirodela polyrhiza.* The size of *Spirodela polyrhiza* genome is 158 Mb, with 15.79% repeats. It possesses 19,623 protein-coding genes, which is 28% lower than those in *Arabidopsis thaliana* (a dicotyledonous plant) and 50% lower than those in rice (a monocotyledonous plant). It is the smallest monocotyledon genome, which serves as a valuable genetic resource to investigate the evolution of monocotyledon. Nonetheless, the 158 Mb genome sequence of *Spirodela* has not resolved to chromosomes; meanwhile, the vital genome features are not determined yet. Therefore, Michael et al. [41] performed rapid whole-genome physical mapping and high-coverage short-read sequencing for the resolution of 20 *Spirodela* chromosomes. They overcame these limitations to obtain genome-wide information on intraspecific variations between different *Spirodela* populations. *Lemna minor* is a model system of aquatic plants for ecotoxicological bioassays, genetic transformation tools, and industrial applications—its whole genome information needs to be studied. Hoeck et al. first reported the *Lemna minor* genome in 2015 [42], which is 472 Mb with 22,382 protein-coding genes with 61.5% repeated sequences. This genome illustrates that the proteins related to biosynthetic processes in response to diverse hydrolase activities are enriched in the *Lemna* compared with the *Spirodela*. Next, Evan Ernst from Rob Martienssen’s group (Cold Spring Harbor Laboratory, USA) performed genome sequencing of *Lemna gibba 7742a* and *Lemna minor 8627* [43,44], whereas Hoang et al. [45] performed genome sequencing of eleven duckweed species from five genera. The sequencing revealed that the duckweed genome size ranges from 160 Mb in *Spirodela polyrhiza* to 2203 Mb in *Woffia arrhiza*, and the largest genome size variation was observed in *Wolfia* (from 432 to 2203 Mb). Except for two *Spirodela* species, the genome size of other duckweeds was 26% higher than that reported by Wang et al. [46]. Recently, An et al. [47] re-sequenced the genome of *Spirodela polyrhiza* using the PacBio long-read sequencing platform. Contig N50 increased by 44 times to 831 kb, while covering the 95.4% gap in the previous *Spirodela polyrhiza* genome data. The sequence information obtained by long-read sequencing explains the evolution and adaptability of the duckweed to the environment. In conclusion, genomic studies may help to develop transgenic duckweeds for phytoremediation, and bioenergy and biomass production. The genome information of duckweed species reported is summarized in Table 2.

## 4. Advantages of the Duckweed Platform

**Figure 3:**
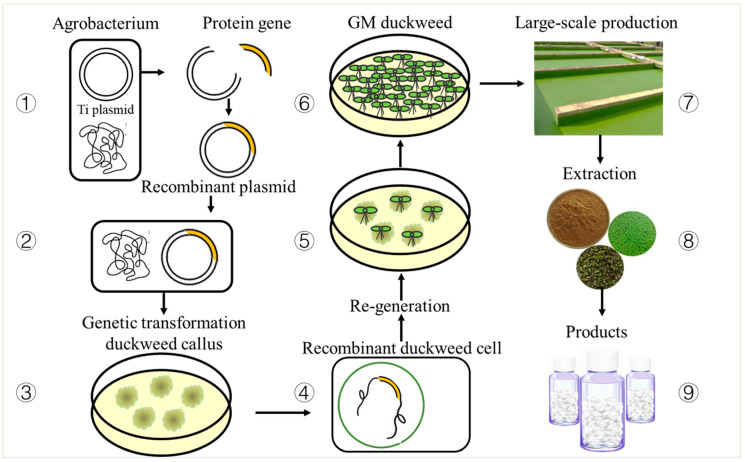
Duckweed platform. The schematic diagram shows the general process of using duckweed as a target products expression platform: ① Clone the target gene and integrate it to obtain a recombinant plasmid. ② Transform the recombinant plasmid into a suitable *Agrobacterium tumefacien*. ③ Co-culture the recombinant *Agrobacterium tumefacien* with duckweed callus. ④ Obtain recombinant duckweed cells using a selective medium. ⑤ Induce recombinant duckweed cells to regenerate frond. ⑥ Identify and reproduce stable and genetically modified duckweed. ⑦ Apply the stable and uniform transgenic duckweed for large-scale production. ⑧ Extract the target protein after cultivation to obtain biomass. ⑨ Purify the target protein and process it for product development.

Duckweed can be used as a platform for bioproduction because of the following advantages over other plants: (1) Duckweed is small in size and can absorb nutrients from water through their fronds or roots and can also secrete target proteins directly into the culture medium [48]. (2) Duckweed has a fast growth rate, usually with a doubling time of 1 to 2 days in a suitable environment [15]. (3) Duckweed adapts to a wide pH and temperature range and can grow rapidly under relatively simple environmental conditions with low production costs [49]. (4) Duckweed shows high dry biomass/unit time and crude protein content (Table 3) with a high nutritional and medicinal value, which can be used in fodder processing, new energy development, and pharmaceutical manufacturing. Stomp et al. predicted that using duckweed to produce medicinal proteins would bring considerable benefits [16]. Inevitably, there must be some logistical concerns to growing hectares of duckweed. This is a common problem for all plants, not just duckweed, and building plant factories is a feasible solution. (5) During the production of recombinant proteins for medicinal purposes, the animal expression system may get contaminated by animal pathogens; however, duckweed offers easy access to sterile pure culture without microbial and chemical pollutants [50]. This feature greatly facilitates reducing the contamination of products and thus has higher safety compared with the animal-based expression system [16]. Therefore, duckweed is an ideal synthetic biology “chassis plant”. Figure 3 shows the process of obtaining target products using duckweed.

## 5. Genetic Transformation System for Duckweed

### 5.1. Duckweed Tissue Culture

The establishment of a complete and efficient plant tissue culture technology is the basis for the genetic transformation of duckweed. Studies on tissue culture of duckweed have been performed since the 1870s. Chang and Chiu [52] first reported and established the tissue culture method for *Lemna gibba*. They found that conjunction of N6-(2-Isopentenyl) adenine (2IP) (1 mg/L) and 2,4-dichlorophenoxyacetic (2,4-D) (10 mg/L) effectively induced callus formation, and the addition of indole acetic acid (4 mg/L) and kinetin (1 mg/L) successfully induced frond regeneration. Subsequently, the tissue culture of a wide range of *Lemna* was established, including *Lemna aequinotialis* [53], *Lemna minor* [54,55], and *Lemna turionifera* [56]. Li et al. [57] established an efficient tissue culture cycle for *Spirodela oligorrhiza*, *Landoltia punctata*, and *Lemna gibba*. Huang et al. [58] investigated known and unknown influencing factors for callus induction of *Landoltia punctata*, and an induction rate of nearly 100% was achieved within 30 days. The best medium to achieve callus induction was the Murashige and Skoog (MS) basal medium containing 15 mg/L 2,4-D, 1% sorbitol, and 2 mg/L 6-BA. Meanwhile, the MS medium that contained 1.0 mg/L 6-BA, 0.5% sucrose, and 1% sorbitol was the best medium for plant regeneration, and a 90% frond regeneration rate was achieved. Khvatkov et al. [59] established a two-step method for *Woffia arrhiza* tissue culture that boosted the callus formation, and the frond regeneration rate increased up to 97% and 78%, respectively. The same conditions were also successfully employed for inducing callus and regeneration of *Woffia globosa* [60].

The tissue culture of four genera of duckweed has been reported (Table 4); however, the induction conditions for different duckweed species were completely different. Fortunately, there still exist certain patterns to study. At the stage of callus induction, as for basal medium, MS was suitable for most *Spirodela*, *Landoltia*, and *Lemna* species, whereas Schenk and Hildebrandt (SH) was more suitable for *Woffia*. As for sugar resources, *Spirodela* and *Landoltia* preferred sorbitol, while *Lemna* and *Woffia* preferred sucrose. For supplements, 2,4-D was used for almost all genera of duckweed, 6-BA was used for some *Spirodela*, *Lemna*, and *Woffia* species, and thidiazuron (TDZ) was only used for certain *Lemna* species. In addition, p-Chlorophenoxy acetic acid (PCA), Dicamba (Di), or 2IP was added to certain species. At the stage of plant regeneration, the same basal media with callus induction stage were always used, except for *Landoltia*. As for supplements, indole-3-acetic acid (IAA) and kinetin (KT) were feasible for most *Lemna,* whereas *Woffia* could be regenerated with a regulator-free basal medium. TDZ, 2,4-D, 2IP, or N6-Benzyladenine (6-BA) was effective in *Spirodela* and *Landoltia*. These basic rules greatly reduced the time we spend in standardizing the tissue culture conditions.

### 5.2. Duckweed Transformation System

The genetic transformation system is an effective approach to combine theory and application, and plays an essential role by using duckweed as a bioreactor to produce recombinant proteins. Two main genetic transformation systems are used in duckweed: The callus transformation system (CTS) and the frond transformation system (FTS) [55].

In 2008, Rival et al. [62] established the *Agrobacterium*-mediated genetic transformation system for multiple strains of *Spirodela polyrhiza*, in which one synthetic gene encoding the mature aprotinin sequence was obtained together with a CaMV 35S promoter-driven secretory signal peptide, and a total of 25 strains of *Spirodela polyrhiza* were transformed. In 2011, Chhabra et al. [54] co-cultured *Lemna minor* callus with *Agrobacterium* EHA105 and selected the transgenic cells using the kanamycin. β-glucuronidase (GUS) active histochemical analysis, polymerase chain reaction (PCR), and Southern hybridization confirmed the transgenic plants. After 11–13 weeks, the whole plant was transformed and used to produce medicinal proteins. Firsov et al. [63] studied the CTS of *Lemna minor* and successfully established the relevant transformation system with an efficiency of 82.5%. Canto-Pastor et al. [64] established the callus transformation system of *Lemna gibba* G3 and *Lemna minor* 8627 with an efficiency of 59%. In 2018, Yang et al. [56] established a *Spirodela polyrhiza* 5543 callus transformation system with an efficiency of 13%. In 2019, Kozlov et al. [65] transformed the callus of *Lemna minor* with *Agrobacterium*-meditated, and eight hirudin and seven glucuronase transgenic lines were obtained.

As the process of callus transformation is time-consuming, has limited genotypes, and requires callus culture and regeneration, it is unsuitable for many dominant duckweed plants for bioreactor development. Kruse et al. [66] used FTS for the transient transformation of *Woffia columbiana* by particle bombardment. Ko et al. [67] established FTS for *Lemna minor,* however, the efficiency of this system was not studied. Khvatkov et al. [68] established FTS for *Woffia arrhiza* based on *Agrobacterium*-mediated transformation. Stable transformed *Wolffia* strains were obtained under the established *Agrobacterium* transformation conditions, and the transgenic efficiency of 100 explants was ~0.2%–0.4%. Yang et al. [55] established FTS for *Lemna minor* as suggested by PCR, quantitative PCR, and GUS-staining results, together with additional detection. FTS was able to acquire steady transgenic lines. Compared with CTS, FTS avoided the genotype restriction during callus induction that also saved around half of the processing time.

## 6. Synthetic Products of Duckweed

Using a genetic transformation system and expression system, a variety of exogenous proteins have been successfully expressed in duckweed (Table 5).

### 6.1. Vaccine Antigens

Transgenic plants serve as novel bioreactors for the production of vaccines and integrate immune response with genetic engineering in plants. Recent studies have reported that the antigen-specific protein can be detected in various plant tissues, humans, and animals. Compared with conventional vaccines, those derived from plants are advantageous because of the safety and cost-effectiveness associated with them. Ko et al. [67] assessed the feasibility of expressing the protective antigen of porcine epidemic diarrhea virus (PEDV) using *Lemna minor*. PCR, Real-timePCR, and Western blotting were performed to verify the transgenic lines. They first detected an anti-animal infectious disease vaccine antigen from duckweed, providing insights for the industrial development of edible antibodies. Firsov et al. [63] successfully expressed the M2e gene of avian influenza virus (H5N1) in transgenic *Lemna minor* to develop an avian influenza vaccine. The crude protein extracts were collected for quantitative enzyme-linked immunosorbent assay (ELISA), and m130-B-glu-curonidase accumulation was found to be 0.09–0.97 mg/g fresh weight (FW) (equal to 0.12%–1.96% of the total soluble protein), which was similar to the values obtained in transient viral systems and can be considered further for the development of an edible plant-produced avian influenza vaccine. They also studied whether it was feasible to express the fusion protein (M2e fused to the ricin toxin B chain, RTB–M130) in duckweeds with nuclear transformation and assessed the immunogenicity of the fusion protein. Their results suggested that duckweed can be successfully transformed using *Agrobacterium*, and peptide M2e was expressed as a part of the RTB–M130 within the transgenic plant. Additionally, they proved the immunogenicity of the incompletely purified transgenic plant-derived total protein at the time of ingestion, which was found to activate the immune response in experimental mice [69]. In 2018, Bertran et al. [70] successfully expressed a hemagglutinin (HA) synthesis gene from H5N1 avian influenza virus in *Lemna minor* and concluded that HA expressed in *Lemna minor* had immunity to resist homologous attacks and is protected against heterologous attacks, which was consistent with the results obtained from the inactivated whole-virus vaccines. HA derived from transgenic duckweed can be used for the production of high-quality vaccine antigens against the H5N1 HPAI virus. Recently, Heenatigala et al. [71] amplified the *LamB* gene from virulent *Vibrio alginolyticus* and introduced it into *Woffia globosa* by *Agrobacterium*-mediated callus transformation [60]. The transgenic duckweed was evaluated by orally vaccinating zebrafish, resulting in high relative percent survival of the vaccinated fish (63.3%).

### 6.2. Therapeutic Products

Until now, a few vital medicinal proteins have been successfully expressed in duckweeds, such as human growth hormone, anti-interferonα-2, hemagglutinin, human granulocyte colony-stimulating factor, monoclonal antibody (mAb), and porcine epidemic diarrhea virus (PEDV) protective antigen.

In 2001, BIOLEX Therapeutics produced bioactive recombinant polypeptides in duckweed plant culture or duckweed root tumor culture. The stable transformed duckweed expressed the bioactive protein hormone, growth factor or cytokine, insulin, or human growth hormone (especially human growth hormone). Among them, the production of interferon-α2 reached a more than 30% protein level in the medium, and the content of human auxin in the culture medium was 609 mg/L. The content of Fab fragment in the dry weight of duckweeds was 8.62 g/kg, accounting for 4% of the total soluble protein. The content of the mAb protein in the dry weight of duckweeds was 5.6 g/kg, accounting for 2.8% of the total soluble protein [72]. Cox et al. [73] expressed the human mAb in *Lemna minor*, which can be used further for the production of therapeutic protein without zoonotic pathogens. In this study, the glycosylation of mAb was optimized by RNA interference during the co-expression of heavy and light strands of mAb. Relative to Chinese hamster ovary cells-expressed monoclonal antibodies cultivated in vitro, the superior effector cell receptor binding activity was observed in mAbs, together with cell-mediated cytotoxicity. Apart from safety and simplicity, the *Lemna minor* expression system also possesses improved antibody functionality, production consistency, and glycosylation homogeneity. Khvatkov et al. [74] reported the expression of human granulocyte colony-stimulating factor (G-CSF) within the nuclear transformed *Woffia arrhiza*. The G-CSF nucleotide sequence was improved as a result of the expression in *Woffia arrhiza*, and was then cloned into the pCamGCSF downstream vector for the CaMV 35S double promoter. According to Western blotting and quantitative ELISA results obtained for these strain-derived protein extracts, the target protein was accumulated in 33 transgenic lines. Based on these findings, the fresh weight of *Woffia arrhiza* contained 35.5 mg/L G-CSF (equal to 0.194% of the total soluble protein). This high production of the recombinant protein is promising for the development of *Wolffia*-related expression systems that have closely regulated formats to generate various recombinant proteins.

### 6.3. Industrial Enzymes

Spencer et al. [75] used the duckweed expression system to produce high levels of microplasminogen along with plasminogen, both of which might generate polypeptides upon activation with protease activity. Rival et al. [62] identified a synthetic gene that encoded the mature aprotinin sequence together with a CaMV 35S promoter-mediated secretory signal peptide by transforming *Spirodela oligorrhiza*. A total of 25 strains of *Spirodela oligorrhiza* were identified by Northern and Western blotting, and the production of aprotinin in the strain was confirmed. Aprotinin levels as high as 3.7% of water-soluble proteins in plants and 0.65 mg/L in growth media were detected. As verified by determining the amino acid sequences and purified by immunoaffinity, aprotinin was correctly spliced in the duckweed expression system and secreted into the growth medium. Sun et al. [76] expressed endonuclease E1 in eosinophilic digestive cells of transgenic *Lemna minor* 8627 without any phenotypic effect on its morphology or growth rate. The E1 enzyme expressed by duckweed had the biological activity, with an optimal temperature of 80 °C, optimal pH of 5, and an expression of 0.24% of the total soluble protein. This study indicated that duckweed may act as a new choice for cellulase expression in transgenic plants. Kozlov et al. [65] recently studied the expression of β-glucuronidase and hirudin in *Lemna minor* L. with *Agrobacterium*-mediated transformation. They acquired eight hirudin-transformed and seven β-glucuronidase gene-transformed transgenic plant lines. Results of histochemical staining and Western blotting confirmed the glucuronidase gene expression. As analyzed by transgenic plant ELISA, β-glucuronidase levels in these transgenic plant lines were 0.28% to 1.43% of the total soluble protein. The results of RT-PCR assay verified hirudin-1 gene expression, and the greatest hirudin accumulation was equivalent to 0.02% of the total soluble protein. Therefore, duckweed can be adopted to develop an expression system for obtaining recombinant proteins such as hirudin in the pharmaceutical field.

**Table 5:** Summary of duckweed synthesis products.

Species	Transformation System	Promoter	Product	Yield	Refs
*Lemna minor*	FTS	CaMV 35S	porcine epidemic diarrhea virus (PEDV)	can be detected by Western blotting	[67]
*Lemna minor*	CTS	CaMV 35S	M2e gene of avian influenza virus (H5N1)	0.09–0.97 mg/g (FW) 0.12–1.96% (TSP)	[63]
*Lemna minor*	CTS	CaMV 35S	M2e fused to the ricin toxin B chain (MRTB–M130)	0.25–2.5 µg/g (FW) 0.0006–0.01% (TSP)	[69]
*Lemna minor*	__	__	hemagglutinin (HA)	__	[70]
*Woffia globosa*	CTS	CaMV 35S	LamB	can be detected by immunoblot	[60,71]
*Lemna minor*	CTS	mas	monoclonal antibody (mAb)	2.1% (TSP)	[18,73]
*Lemna minor*	CTS	mas	human growth hormone	609 mg/L (culture medium)	[72]
*Lemna minor*	CTS	mas	interferon-α2	30% (protein content of culture medium)	[72]
*Lemna minor*	CTS	mas	fragment of antigen binding (Fab)	8.62 g/kg (DW) 4% TSP	[72]
*Woffia arrhiza*	CTS	CaMV 35S	human granulocyte colony-stimulating factor (G-CSF)	35.5 mg/kg (FW) 0.194% (TSP)	[74]
*Lemna minor*	CTS	mas	plasminogen	6.4% (TSP)	[75]
*Lemna minor*	CTS	mas	microplasminogen	290 ng/mL (culture medium)	[75]
*Spirodela oligorrhiza*	CTS	CaMV 35S	aprotinin	0.65 mg/L (culture medium) 3.7% (water soluble proteins)	[62]
*Lemna minor*	CTS	CaMV 35S	Endoglucanase E1	Up to 0.24% (TSP)	[18,77]
*Lemna minor*	CTS	CaMV 35S	β-glucuronidase	0.28 to 1.43% (TSP)	[65]
*Lemna minor*	CTS	CaMV 35S	hirudin	0.02% (TSP)	[65]

FTS, Agrobacterium-mediated fronds transformation; CTS, *Agrobacterium*-mediated callus transformation; FW, fresh weight of duckweed; DW, dry weight of duckweed; TSP, the total soluble protein; Refs: references.

## 7. Conclusions and Prospects

After Barbara Hoppen first proposed “synthetic biology” to describe the genetically engineered bacteria in 1980 [77], a variety of natural products and their precursors have been achieved in microorganisms. Plants, multicellular organisms, possessing abundant inner membrane systems, and organelles with complex temporal and spatial characteristics, provide the necessary foundation for the synthesis of numerous enzymes and metabolites [78]. A plant bioreactor is a plant cell or whole plant processed by transgenic engineering to produce biological derivatives or biological products with a high added value for multiple uses [9]. Previous studies have reported several incidents of the release of genetically modified organisms (GMOs) and contamination of food supply. Therefore, the control of GMO products is an important challenge for utilizing the plant gene expression system. It not only leads to food crisis but also restricts industrialization. Therefore, plants with easy cultivation, simple genetic background, high yield, and low cost should be selected [79]. Duckweeds are an ideal “chassis plant” for synthetic biology, and they provide a promising bioproduction platform to produce polymers, proteins, and small molecules [16,72].

In recent years, duckweed has shown potential in biosynthesis to act as a bioreactor, with advantages such as high protein yield and stable storage of target proteins. It can be used as a biosynthesis platform for the production of vaccines, antibodies, pharmaceutical proteins, and industrial enzyme preparations. An in-depth study of the whole-genome sequencing of duckweeds will help to understand the biology of duckweed species and to use them for producing biomass. The establishment of the complete plant tissue culture system and the genetic transformation system of duckweed effectively combine the theory and application, enabling the rapid development of duckweed synthetic biology. Gene engineering technology has been widely used in the synthetic biology of duckweed. Finally, several researchers have successfully integrated multiple exogenous protein genes into the duckweed genome and made it to express corresponding target products, which is a breakthrough in the biosynthesis of duckweed as a new bioreactor.

To sum up, duckweed is a tiny aquatic angiosperm with great application prospects, which can be regarded as a new bioreactor suitable for various studies and applications. With further improvement in genome sequencing and the genetic transformation system of duckweed, more biological products will be produced by using genetic engineering of duckweed.

## Figures and Tables

**Table 1:** Duckweed chloroplast genomes assembly results.

Genera	Species	CGS (bp)	IRs (bp)	GN	References
*Spirodela*	*Spirodela polyrhiza 7498*	168,956	31,844	MN419335	[33]
*Landoltia*	*Landoltia punctata ZH0202*	171,013	31,899	KY993962	[32]
*Lemna*	*Lemna minor*	165,955	31,223	DQ400350	[30]
*Woffia*	*Woffia australiana 7733*	168,704	31,930	JN160605	[31]
*Woffiella*	*Woffiella lingulata 7289*	169,337	31,683	JN160604	[31]

CGS, chloroplast genome size; IRs, inverted repeats; GN, GenBank number.

**Table 2:** Complete duckweed genomes.

Species	Genome Size (Mb)	Platform	Sequencing Coverage	Protein Coding Gene	Scaffold N50	Contig N50 (Kb)	Repeat (%)	References
*Spirodela polyriza 7498*	158	454 and Sanger	21	19,623	3.8 Mb	18	17	[40]
*Spirodela polyriza 9509*	160	Illumina and BioNano	95	18,507	7.6 Mb	19	23.8	[41]
*Lemna minor 5500*	481	Illumina	120	22,382	23.6 Kb	20.9	61.5	[42]
*Lemna minor 8627*	800	Illumina and PacBio	__	__	__	222	__	[43]
*Lemna gibba 7742a*	450	Illumina	__	21,830	520 Kb	53	__	[43]

**Table 3:** Comparison of protein yield between duckweed and high-protein crops [51].

Plant	Duckweed	Soybean	Peanut	Alfalfa
Dry biomass (t/ha/year)	17.6	1.59	1.6–3.12	4.37–15.69
Crude protein content (%)	37	42	23.6	15.9–17

**Table 4:** Tissue culture of duckweed.

Genera	Species	Callus Induction		Plant Regeneration		Refs
		BM	Supplements (mg/L)	BM	Supplements (mg/L)	
*Spirodela*	*Spirodela oligorrhiza SP*	WP + 2% So + 1% Ma	PCA (5) + 2IP (2)	WP + 0.5% Su	TDZ (1)	[57]
	*Spirodela polyrhiza 5543*	1/2 MS + 1% So	2,4-D (5) + 6-BA (2)	1/2 MS + 1% So	2,4-D (5) + 6-BA (2)	[61]
*Landoltia*	*Landoltia punctata 8717*	1/2 MS + 1% So	2,4-D (3.5) + Di (15) + 2IP (2)	WP + 0.5% Su + 1% So	2IP (1)	[57]
	*Landoltia punctata 5502, 8721, and 9264*	MS + 1% So	2, 4-D (15) + 6-BA (2)	MS + 0.5% Su	6-BA (1)	[58]
*Lemna*	*Lemna gibba G3*	MS + 3% Su	2, 4-D (10) + 2IP (1)	MS + 3% Su	IAA (4) + KT (1)	[52]
	*Lemna gibba var. Hurfeish*	B5 + 1% Su	Di (50) + 6-BA (15)	B5 + 1% Su	TDZ (1)	[57]
	*Lemna aequinotialis 6002*	MS + 3% Su	2, 4-D (1) + TDZ (0.1)	B5 + 1% Su	IAA (0.45) + KT (1)	[53]
	*Lemna minor*	B5 + 1% Su	2, 4-D (11) + TDZ (1.1)	B5 + 1% Su	IAA (4.4) + KT (1.1)	[54]
	*Lemna minor ZH0055*	MS + 3% Su	2, 4-D (10) + TDZ (5)	MS + 3% Su	IAA (4.4) + KT (1.1)	[55]
	*Lemna turionifera 5511*	B5 + 1.5% Su	2,4-D (1) + Di (15) + 6-BA (1)	B5 + 1.5% Su	L-serine (105)	[56]
*Woffia*	*Woffia arrhiza* (L.) *Horkel ex Wimm*	SH + 2% Su	2, 4-D (5) + 6-BA (0.5)	SH + 2% Su	regulator-free	[59]
	*Woffia globosa*	SH + 2% Su	2, 4-D (5) + 6-BA (0.5)	SH + 2% Su	regulator-free	[59,60]

BM, basal medium; WPM, woody plant medium; MS, Murashige and Skoog medium; SH: Schenk and Hildebrandt medium; B5, Gamborg medium; PCA, p-Chlorophenoxy acetic acid; 2IP, N6- (2-Isopentenyl) adenine; TDZ, thidiazuron; 2,4-D, 2,4-dichlorophenoxyacetic; 6-BA, N6-Benzyladenine; IAA, indole-3-acetic acid; KT, kinetin; Ma, Mannitol; So, sorbitol; Su, sucrose; Di, Dicamba, 3,6-dichloro-2-methoxybenzoic; Refs: references.

## Data Availability

Not applicable.
